# Effect of oral melatonin and wearing earplugs and eye masks on nocturnal sleep in healthy subjects in a simulated intensive care unit environment: which might be a more promising strategy for ICU sleep deprivation?

**DOI:** 10.1186/s13054-015-0842-8

**Published:** 2015-03-19

**Authors:** Hua-Wei Huang, Bo-Lu Zheng, Li Jiang, Zong-Tong Lin, Guo-Bin Zhang, Ling Shen, Xiu-Ming Xi

**Affiliations:** Department of Critical Care Medicine, Fuxing Hospital, Capital Medical University, 20A Fu Xing Men Wai Da Jie, Xicheng District, Beijing, 100038 P.R. China; Department of Pediatric Surgery, Fuzhou Children’s Hospital of Fujian Province, Teaching Hospital of Fujian Medical University, Fuzhou, Fujian 350005 P.R. China; Department of Otorhinolaryngology, Fuzhou Children’s Hospital of Fujian Province, Teaching Hospital of Fujian Medical University, Ba Yi Qi Zhong Road, Gulou District, Fuzhou, Fujian 350005 P.R. China; Department of Neurosurgery, Beijing Tiantan Hospital, Capital Medical University, Tiantan Xili 6, Chongwen District, Beijing, 100050 P.R. China

## Abstract

**Introduction:**

Sleep deprivation is common in critically ill patients in the intensive care unit (ICU). Noise and light in the ICU and the reduction in plasma melatonin play the essential roles. The aim of this study was to determine the effect of simulated ICU noise and light on nocturnal sleep quality, and compare the effectiveness of melatonin and earplugs and eye masks on sleep quality in these conditions in healthy subjects.

**Methods:**

This study was conducted in two parts. In part one, 40 healthy subjects slept under baseline night and simulated ICU noise and light (NL) by a cross-over design. In part two, 40 subjects were randomly assigned to four groups: NL, NL plus placebo (NLP), NL plus use of earplugs and eye masks (NLEE) and NL plus melatonin (NLM). 1 mg of oral melatonin or placebo was administered at 21:00 on four consecutive days in NLM and NLP. Earplugs and eye masks were made available in NLEE. The objective sleep quality was measured by polysomnography. Serum was analyzed for melatonin levels. Subjects rated their perceived sleep quality and anxiety levels.

**Results:**

Subjects had shorter total sleep time (TST) and rapid eye movement (REM) sleep, longer sleep onset latency, more light sleep and awakening, poorer subjective sleep quality, higher anxiety level and lower serum melatonin level in NL night (*P* <0.05). NLEE had less awakenings and shorter sleep onset latency (*P* <0.05). NLM had longer TST and REM and shorter sleep onset latency (*P* <0.05). Compared with NLEE, NLM had fewer awakenings (*P* = 0.004). Both NLM and NLEE improved perceived sleep quality and anxiety level (*P* = 0.000), and NLM showed better than NLEE in perceived sleep quality (P = 0.01). Compared to baseline night, the serum melatonin levels were lower in NL night at every time point, and the average maximal serum melatonin concentration in NLM group was significantly greater than other groups (*P* <0.001).

**Conclusions:**

Compared with earplugs and eye masks, melatonin improves sleep quality and serum melatonin levels better in healthy subjects exposed to simulated ICU noise and light.

**Trial registration:**

Chinese Clinical Trial Registry ChiCTR-IPR-14005458. Registered 10 November 2014.

## Introduction

Sleep deprivation is a major concern in critically ill patients in the ICU, and is characterized by poor subjective sleep quality, a paucity of restorative sleep stages and loss of circadian rhythms. [1] Because of frequent arousals and awakenings, sleep in the ICU has a higher proportion of none-rapid eye movement (NREM) sleep stage 1 and 2 (or light sleep), and reduced restorative slow wave (SW) and rapid eye movement (REM) sleep [2], and might bring many adverse consequences, including impaired immune function, difficult weaning from mechanical ventilation, delirium and severe morbidity [3].

The cause of sleep disturbance in the ICU is multifactorial. Aside from the primary diseases, medications, mechanical ventilation and so on, ICU noise and light environment, and hormonal imbalance play essential roles [4-8]. Gabor *et al*. reported that ICU noise and patient care activities were responsible for up to 30% of the arousals and awakenings in ICU patients [9]. Some studies also found that ICU noise or/and light might increase the sleep-onset latency, shorten total sleep time and disturb sleep structure in healthy subjects [6,10,11]. Hu *et al*. suggest that ICU noise and light might disturb sleep by suppressing nocturnal melatonin levels [6]. Therefore, the ICU environment is a critical factor that causes ICU sleep disturbance, which can be relatively easy to control and diminish, in contrast to most other factors.

Currently, there are many strategies for ICU acousto-optic control, such as controlling noise and dimming the light at night and large-scale ICU environmental reform and so on, but the low universality and poor feasibility are the major problems [12]. Therefore, some domestic and international experts recommend that ICUs incorporate earplugs/eye masks into routine nursing care: however, effectiveness is still a matter of debate [13]. Hu *et al*. found earplugs and eye masks might elevate the nocturnal melatonin level when tested in healthy subjects in a simulated ICU environment [6]. However, most critically ill patients in the ICU lose their ability to regulate melatonin secretion by exposure to darkness and light. [14] Therefore, more effective strategies for improving sleep disruption induced by ICU noise and light are urgently needed.

Melatonin is a substance with pleiotropic physiologic action synthesized in the pineal gland [15]. Its secretion is suppressed by light and stimulated by darkness [15]. The disruption to the normal timing and amplitude of the circadian rhythm of melatonin secretion is associated with disturbed sleep [16,17]. Exogenous melatonin has been demonstrated to be safe and effective in the treatment of primary insomnia in the elderly and other circadian rhythm sleep disorders [18-22]. Recently several studies found melatonin levels in critically ill patients to be severely depressed [23-27], which raised interest in melatonin as a potential therapeutic or prophylactic agent in the management of ICU sleep disturbance. However, until now the influence of melatonin treatment on sleep quality in ICU patients remained controversial [28-30]. No studies have yet evaluated the effects on sleep in ICU patients of oral melatonin, as measured by polysomnography (PSG), the gold standard for assessing sleep quality. However, critically ill patients have disturbed electroencephalographic patterns caused by many complex factors as seen in sepsis, neurologic pathology and medication, among others, and the sleep staging in accordance with the American Association of Sleep Medicine 2007 criteria cannot be met [31]. Therefore, in our study we recruited healthy participants as experimental subjects and conducted the research in a simulated ICU environment, and adopted the gold standard assessment technique to verify our hypothesis. Meanwhile, the comparative study of different strategies for addressing the problem of ICU sleep disturbance has not been performed. In this study, we aimed to investigate and compare the effectiveness of oral melatonin, and wearing of earplugs and eye masks, on sleep quality in healthy subjects who were exposed to a simulated ICU situation.

## Materials and methods

### Study design and participants

The study protocol was formally approved by the Institutional Review Board of Fuzhou Children’s Hospital (approval number 2014–001) and by the Chinese Clinical Trial Registry (approval number ChiCTR-IPR-14005458). The study was carried out in accordance with the Declaration of Helsinki principles. All participants provided written informed consent. Subjects were included in the study if they were older than 18 years of age, had body mass index (BMI) <26 kg/m_2_, were non-smokers, had scores ≤7 on the Pittsburgh sleep quality index (PSQI), had almost had no daytime sleep and slept only at night, that is, they went to bed between 21:00 and midnight and habitually spent in between 6 and 9 h per night in bed. Exclusion criteria included a history or current diagnosis of other sleep disorders (such as restless leg syndrome, periodic leg movements with arousals, narcolepsy, REM behavior disorder, circadian rhythm sleep disorder, breathing-related sleep disorder, or parasomnia), which was assessed by the clinical manifestation and a diagnostic PSG record (which was also performed to familiarize subjects with the PSG procedures); reduced hearing acuity (>20 dB hearing loss at a single frequency, as tested with an audiometer (Entomed SA 201); blindness (as tested with visual testing and perimetry), and a history of alcohol or medication abuse. Participants with an occupational history that included shift work or recent significant travel across three or more time zones within the prior two weeks were also excluded. In addition, after a screening PSG, participants with an apnea-hypopnea index >15 or a periodic leg-movement arousal index >15, and known allergy to melatonin, were also excluded.

**Figure 1:**
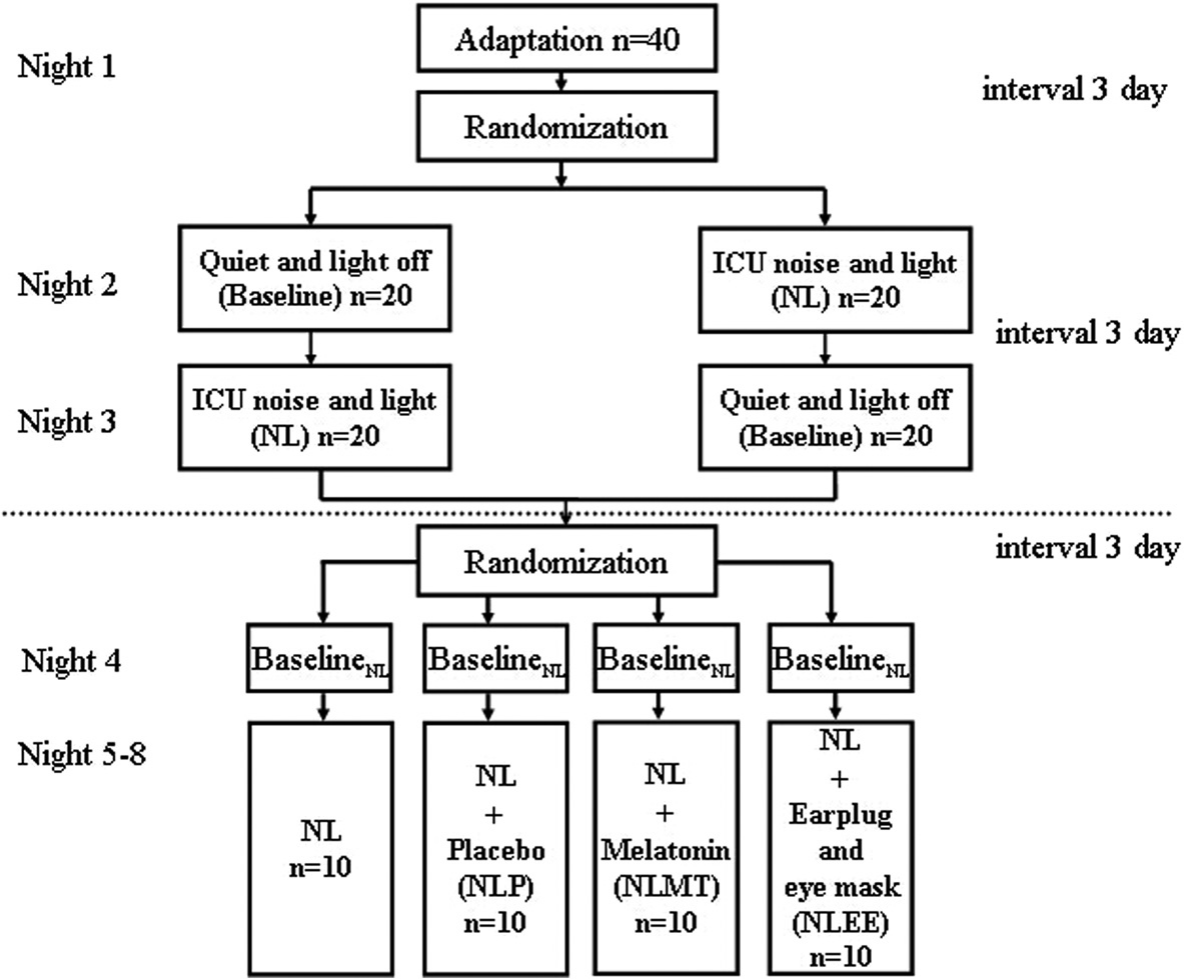
**Study design.**

All healthy participants (n = 40) slept in individual private rooms for eight nights (21:00 to 06:00) (Figure 1). The first night served as adaptation, that is, the participants followed the same procedure and data (not to be used in the analyses) were collected just as in the following nights. Then, the study was conducted in two stages. The first stage used a crossover design to investigate the impact of ICU noise and light environment on the sleep quality of healthy subjects. To minimize order effects, half of the healthy subjects (n = 20) were randomly exposed to a simulated ICU noise and light (NL) environment on the second night and to a quiet and dark environment (baseline) on the third night. In the meantime, other participants (n = 20) were exposed in the opposite order. In this stage, all subjects underwent two overnight PSG examinations on the second and third nights. For each subject, study nights were spaced 3 days apart to avoid delay effects. The second stage was to evaluate the effect of melatonin, and earplugs and eye masks, on the sleep quality of healthy subjects exposed to simulated nocturnal ICU noise and light. These 40 participants were assigned randomly to either: (1) simulated ICU noise and light (NL); (2) NL plus placebo (NLP); (3) NL plus melatonin (NLM); or (4) NL plus use of earplugs and eye masks (NLEE) in a 1:1:1:1 ratio.

Randomization was performed using a computer-generated schedule independent of treatment personnel. Subjects in the melatonin and placebo groups did not know they were receiving active therapy, nor did their clinicians. As potential chronophypnotic benefits of melatonin are not immediate and may take at least 3 days to be released, the process of intervention should take 4 days [28-30]. In order to control for possible effects of baseline values on the outcome variable, the baseline data for the simulated ICU environment (Baseline_NL_) needed to be collected and analyzed before the 4-day intervention. Therefore, in this stage, all participants slept in the simulated ICU noise and light environment during the fourth night, and were exposed to corresponding intervening factors based on their group assignment for the following four consecutive nights (fifth to eight night) in the laboratory, and underwent two nighttime PSG evaluations on the fourth night for the baseline, and on the eighth night for assessment of the outcome variable, respectively.

### Intervention and instruments

#### Baseline night (quiet and dark environment)

The laboratory is constructed so that sounds or vibrations from the surroundings are completely prevented and the background noise level with full ventilation is less than 15 dB (A). The dB(A) means that the sound level is measured by A weighting sound level meter. Mean nighttime light levels in the sleep laboratory measured 5 lux with the light off and the door to the hallway shut. Therefore, on the baseline night, all healthy subjects slept in a quiet environment with the light off.

### Simulated ICU noise and light night (NL night)

#### Simulated ICU noise exposure

The exposure sounds in our study were recorded digitally during a typical weekday night shift (21:00 to 06:00) in the ICU at Fujian provincial hospital and stored on computer for playback in the sleep laboratory. ICU noise was continuously monitored during the night using a sound meter, model AW5610D (AWAI, Hangzhou, China) in the surgical ICU (SICU) environments. The SICU had noise levels far exceeding the 20 dB (A) at nighttime recommended by the Guidelines of the Chinese Association of Critical Care medicine (2006). The mean (standard deviation) noise value in the SICU was 67.1 ± 10.2 dB (A), the peak noise level recorded was 99.7 dB (A), and the minimal noise level recorded was 47.3 dB (A). The sound recording equipment, model ICD-P320 (Sony Inc., Tokyo, Japan), was positioned at the bed of patients receiving mechanical ventilation. Simultaneous sound meter readings were taken to ensure similar noise levels during playback in the sleep laboratory.

#### Simulated ICU lighting conditions

Nighttime illumination in the ICU setting and the sleep laboratory were monitored by a light detector model TES1332 (Taiwantes, Shenzhen, China). In both settings, the main light was provided by fluorescent ceiling lights. The light detector was placed by a patient receiving mechanical ventilation, but not so as to interfere with patient care. Light measurements were taken every hour during the night. In the ICU high mean night light levels ranging between 56.0 and 221.3 lux were maintained. The mean nighttime light level in the sleep laboratory measured 100 lux with the light on, and 5 lux with the light off and the door to the hallway shut. Therefore, the study used 100 lux to simulate the ICU lighting conditions.

During Baseline_NL_, NL, NLM, NLEE and NLP nights, recorded ICU noise was played and the fluorescent lights were turned on in the sleep laboratory. A sound meter was placed at the head of the subject’s bed and the recording time synchronized with the sound meter to ensure playback in a similar range of decibels to that recorded.

#### Earplugs and eye masks (NLEE night)

Subjects were instructed to wear earplugs with a 29-dB noise reduction rating (3 M Corporation, Beijing, China) and eye masks during the NLEE night. Subjects chose from three sizes of eye mask provided, which were 18 × 6 cm, 21 × 8 cm, and 24 × 10 cm, respectively. All participants chose the most suitable size according to their face size. We offered earplugs and eye masks to the subjects at night (from 21:00 to 06:00).

#### Melatonin (NLM)

Participants assigned to the melatonin group were given a 1-mg fast-release oral dose of melatonin (Armonia® Retard 1 mg; Nathura, Montecchio Emilia, Italy) administered at approximately 21:00. Dose changes were not permitted. Melatonin is not a licensed drug in China, and it is sold as a food supplement in a variety of preparations. The product used in this study contains a high-purity melatonin preparation (99.9%). This product has been regularly registered in the list of food supplements of the Italian Ministry of Health (cod. 08 29284 Y).

#### Placebo (NLP)

Participants in this group were treated according to a protocol identical to those receiving active medication. As with melatonin, the placebo was made in the identical formulation, and there were no differences in appearance, smell or flavor between the active and inactive pills.

### Sleep measures and laboratory test

#### Polysomnography

Sleep was assessed by PSG using the Polysmith 2003 sleep acquisition and analysis system (Neurotronics, Gainesville, FL, USA). The standard procedure for sleep measurement described by Rechtschaffen and Kales was followed [32]. Subjects were hooked up for recording of an electroencephalogram (EEG), eye movement, and a submental electromyogram (EMG) in the sleep laboratory. Electrode impedances were within acceptable limits (<10 kQΩ). PSG equipment was located outside the subject’s room. Sleep variables (sleep period time, sleep efficiency index, sleep-onset latency, REM latency, arousal index and percentage of sleep in REM, stage one, two and three and so on) were scored manually and independently by two scorers who were unaware of the experimental conditions, according to standardized criteria. Polysomnographic records were collected from 21:00 to 06:00 on nights 1 to 4 and on night 8.

### Serum melatonin concentration

Nocturnal blood was collected at 20:50 (before administration), 22:00, 23:00, 24:00, 01:00, 02:00, 03:00, 04:00, 05:00 and 06:00 h on nights 2, 3 and 8. In order to avoid repeated venipuncture, it is routine to give all subjects an indwelling vein needle. The trained technicians were required to access the participant’s room as quietly as possible and take blood by the light of an electric torch. Blood samples were collected in plastic tubes without anticoagulant agents and stored at −20°C until assayed. Melatonin concentrations were measured using a commercial radioimmunoassay (RIA) kit for human melatonin (BioSource Europe SA, Belgium). In this assay, sensitivity was 2 pg/mL. The intra-assay and inter-assay coefficients of variation (CV) were 5.6% and 8.2%, respectively.

### Subjective measurements

Subjective sleep quality was assessed by a visual analog scale developed by the researchers based on previous scales [33]. Subjects evaluated their sleep quality on a scale of 0 to 10 (0 = excellent, 10 = poor) at 7:00 am on the morning after nights 2, 3, 4 and 8, with a higher score indicating poorer habitual sleep quality.

State anxiety level was assessed at 7:00 am on the morning after nights 2, 3, 4 and 8. In our study, the Spielberger state anxiety inventory (SAI) was chosen because it provides evaluation of state anxiety levels, namely a temporary unpleasant emotional arousal in the face of threatening demands or dangers. Subjects rated their feelings of anxiety on a 4-point scale ranging from a score of 1 (almost never anxious) to 4 (almost always anxious), a higher score indicating a higher anxiety level.

Subjects were asked to evaluate the comfort, effectiveness and ease of use of earplugs and eye masks on the morning after the NLEE night, using a 5-point scale ranging from a score of 1 (very uncomfortable, very unhelpful, very awkward) to 5 (very comfortable, very helpful, very easy to use), with low scores indicating a less pleasant experience.

### Statistical analysis

Data were analyzed using SPSS version 19.0 (SPSS Inc., Chicago, IL, USA). Data for the adaptation night were excluded from analysis because the first night of sleep in a sleep laboratory room with unfamiliar surroundings differs from sleep on subsequent nights [18]. All data were expressed as mean ± SD. One-way analysis of variance (ANOVA) was used to determine differences in perceived sleep quality and anxiety levels during the four nights of the experiment. The ANOVA for repeated measures can be used to determine differences in sleep variables and melatonin concentrations during the four nights of the experiment. The paired Student *t*-test or non-parametric Wilcoxon rank sum test were performed to evaluate the effect of melatonin, and earplugs and eye masks on sleep variables and melatonin secretion during exposure to simulated ICU sound and light, where appropriate. The chi-square test was used to compare the gender ratio. *P* <0.05 was considered significant.

## Results

Forty healthy subjects were recruited. All 40 subjects (20 female and 20 male, aged 24 to 64 years, mean 41.2 ± 11.8 years) completed the study. The average habitual sleep time of the participating subjects was 7.1 h (SD 0.5 h), with an average habitual time of retiring of 22:05 and of getting up of 06:25. In part 2 of the study, the results were based on the following numbers of subjects: 10 in NL, 10 in NLP, 10 in NLEE and 10 in NLM. Notably, there were no significant differences in the demographic characteristics and clinical variables of the subjects at the baseline of simulated ICU noise and light (Baseline_NL_) for the different study conditions (all *P* >0.05) (Table 1).

**Table 1:** **Characteristics of healthy subjects in the baseline night of simulated ICU noise and light (Baseline**_NL_)

**Variable**	**NI**	**NIP**	**NLEE**	**NLM**	***P***
**Age**	42.8 ± 11.8	39.7 ± 11.6	40.1 ± 12.7	42.0 ± 12.8	0.930
**Gender (percent female)**	0.4	0.5	0.5	0.6	0.849
**Time in bed (min)**	520.6 ± 17.0	509.1 ± 16.4	511.8 ± 17.8	510.9 ± 26.6	0.580
**Total sleep time (min)**	349.2 ± 28.4	354.0 ± 22.6	354.2 ± 20.7	348.6 ± 22.0	0.922
**Sleep efficiency index**	0.69 ± 0.08	0.75 ± 0.08	0.72 ± 0.07	0.75 ± 0.05	0.164
**REM (%)**	16.6 ± 3.6	16.5± 6.3	18.5 ± 3.5	19.3 ± 4.1	0439
**N1 (%)**	17.7 ± 3.9	18.8 ± 3.3	19.6 ± 2.3	19.6 ± 3.2	0.530
**N2 (%)**	47.9 ± 6.3	47.5 ± 7.1	46.2 ± 1.6	43.6 ± 3.4	0.259
**N3 (%)**	15.8 ± 3.5	17.2 ± 4.9	15.7 ± 2.8	17.5 ± 3.5	0.620
**Sleep onset latency (min)**	58.4 ± 15.5	54.3 ± 15.3	53.2 ± 16.9	46.9 ± 11.0	0392
**REM latency (min)**	166.6 ± 38.8	140.4 ± 29.3	159.5 ± 30.0	154.4 ± 37.5	0381
**No. of awakenings**	14.4 ± 3.3	14.4 ± 3.1	13.5 ± 2.8	13.8 ± 3.0	0.885
**Sleep arousals index**	8.7 ± 3.3	8.4 ± 2.4	8.2 ± 1.3	8.4 ± 2.1	0.981
**State anxiety**	46.0 ± 8.8	45.2 ± 7.7	44.4 ± 5.9	45.0 ± 5.9	0.968
**Sleep quality**	6.1 ± 1.0	5.9 ± 1.0	6.1 ± 1.3	6.2 ± 1.4	0.952

NL: simulated ICU noise and light; NLP: NL plus placebo; NLM: NL plus melatonin; NLEE: NL plus use of earplugs and eye masks.

### Sleep architecture

The influence of simulated ICU noise and light on sleep architecture of healthy subjects compared to the baseline night are shown in Table 2. Compared to baseline, total sleep time, sleep efficiency index and the mean percent REM sleep were reduced during the NL night (*P* = 0.000, *P* = 0.001 and *P* = 0.006, respectively). The sleep-onset latency, number of awakenings, sleep arousals index and the mean percent stage 2 non-REM (NREM) sleep were increased during the NL night compared to baseline (*P* = 0.000, *P* = 0.011, *P* = 0.006 and *P* = 0.018, respectively). Mean stages 1 and 3 NREM sleep percentage and REM latency during the night were not different between conditions.

**Table 2:** **Comparison of sleep architecture between baseline night and simulated ICU noise and light night**

**Variable**	**Baseline**	**NL**	***P***
**Time in bed (min)**	507.8 ± 21.1	509.6 ± 23.7	0.860
**Total sleep time (min)**	424.3 ± 25.9	359.2 ± 39.9	0.000
**Sleep efficiency index**	0.83 ± 0.06	0.71 ± 0.08	0.001
**REM (%)**	21.9 ± 3.8	15.8 ± 4.8	0.006
**N1 (%)**	19.6 ± 3.8	19.7 ± 9.8	0.983
**N2 (%)**	39.8 ± 5.1	47.8 ± 8.3	0.018
**N3 (%)**	18.7 ± 5.3	14.7 ± 4.2	0.078
**Sleep onset latency (min)**	23.4 ± 10.1	66.2 ± 20.7	0.000
**REM latency (min)**	147.8 ± 20.1	163.9 ± 55.2	0.399
**No. of awakenings**	9.9 ± 3.7	14.4 ± 3.3	0.011
**Sleep arousals index**	5.0 ± 1.8	8.7 ± 3.3	0.006

Baseline: quiet and dark environment; NL: simulated ICU noise and light.

Results of sleep variables during NL, NLP, NLM and NLEE nights are shown in Table 3. Repeated measures ANOVA showed that sleep architecture changed significantly by condition in the percentage of REM sleep (*P* = 0.05), sleep-onset latency (*P* = 0.001), number of awakenings (*P* = 0.000) and sleep arousals index (*P* = 0.000). Comparison of sleep variables during exposure to the simulated ICU environment indicated that use of earplugs and eye masks resulted in fewer awakenings (*P* = 0.001), shorter sleep-onset latency (*P* = 0.01) and less sleep arousal according to the sleep arousal index (*P* = 0.000). Comparison of sleep variables during exposure to the simulated ICU environment indicated that use of melatonin resulted in more total sleep time (*P* = 0.043), greater percentage of REM (*P* = 0.011), fewer awakenings (*P* = 0.000), shorter sleep-onset latency (*P* = 0.004) and less arousal according to the sleep arousal index (*P* = 0.001). No differences were found between use of earplugs and eye masks, and use of melatonin, in most of sleep variables except the number of awakenings (*P* = 0.004), during exposure to the simulated ICU environment (all *P* >0.05), although the sleep variables showed interesting trends towards better sleep on the NLM night than on the NLEE night.

**Table 3:** **Comparison of sleep architecture in simulated ICU noise and light night for different study conditions**

**Variable**	**NL**	**NLP**	**NLEE**	**NLM**	**ANOVA**	**Contrast 1**	**Contrast 2**	**Contrast 3**
					***P***	***P***	***P***	***P***
**Time in bed (min)**	513.8 ± 18.6	507.3 ± 23.0	507.9 ± 29.7	508.8 ± 26.5	0.934	0.598	0.893	0.936
**Total sleep time (min)**	369.0 ± 35.0	367.6 ± 33.3	385.8 ± 38.9	401.2 ± 35.8	0.137	0.302	0.043	0.342
**Sleep efficiency index**	0.72 ± 0.08	0.72 ± 0.07	0.76 ± 0.08	0.79 ± 0.11	0.186	0.273	0.077	0.396
**REM (%)**	16.0 ± 4.2	15.0± 7.6	18.4 ± 3.7	20.9 ± 3.0	0.050	0.285	0.011	0.258
**N1 (%)**	20.3 ± 8.6	21.1 ± 8.7	20.4 ± 3.2	19.2 ± 4.0	0.928	0.973	0.509	0.681
**N2 (%)**	46.8 ± 7.3	47.4 ± 7.6	46.3 ± 3.0	42.6 ± 3.5	0.262	0.850	0.073	0.161
**N3 (%)**	15.0 ± 3.9	16.5 ± 4.5	14.9 ± 3.1	17.3 ± 3.6	0.425	0.991	0.654	0.175
**Sleep onset latency (min)**	71.4 ± 25.6	61.3 ± 20.6	46.6 ± 21.6	33.7 ± 10.5	0.001	0.010	0.004	0.163
**REM latency (min)**	154.4 ± 46.9	158.2 ± 39.2	158.0 ± 37.4	149.5 ± 37.8	0.959	0.844	0.634	0.644
**No. of awakenings**	15.1 ± 3.3	13.0 ± 3.1	10.5 ± 3.2	6.5 ± 1.8	0.000	0.001	0.000	0.004
**Sleep arousals index**	9.8 ± 3.0	8.2 ± 2.6	5.5 ± 2.1	4.5 ± 1.4	0.000	0.000	0.001	0.392

NL: simulated ICU noise and light; NLP: NL plus placebo; NLM: NL plus melatonin; NLEE: NL plus use of earplugs and eye masks; Contrast 1: NL vs. NLEE; Contrast 2: NLP vs. NLM; Contrast 3: NLEE vs. NLM.

### Serum melatonin level

**Figure 2:**
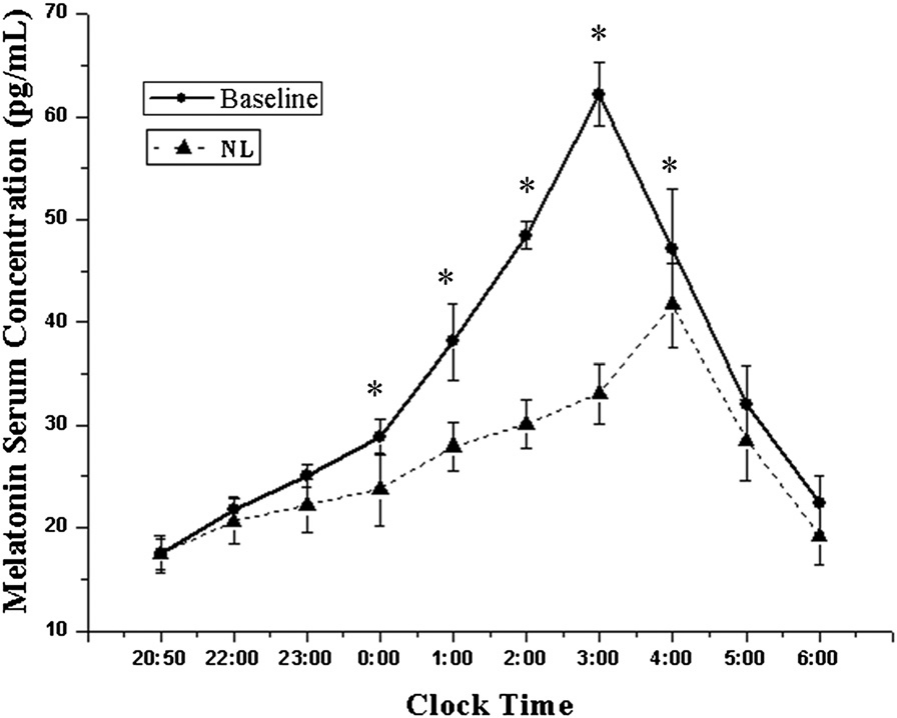
**Melatonin levels in healthy subjects on the baseline night and on the simulated ICU noise and light (NL) night.** Serum melatonin levels were measured in all subjects on baseline and NL nights for 9 h from 20:50 to 06:00. The graph depicts the nocturnal serum melatonin concentration. Points represent mean ± SD. Solid circles, healthy subjects on the baseline night; solid triangles, healthy subjects on the NL night: *P <0.05 at 0:00, 01:00, 02:00, 03:00 and 04:00 for comparison of the baseline and the NL night.

The influence of simulated ICU noise and light on nocturnal serum melatonin levels in healthy subjects compared to the baseline night are shown in Figure 2. Both on baseline and on NL nights, endogenous melatonin secretion followed a similar circadian pattern, with the rise in melatonin levels at around 20:50 (before bedtime), reaching peak concentration at 03:00 and 04:00, respectively, and then gradually dropped. However, compared to the baseline night, the serum melatonin levels were lower on the NL night at every time point. There were significant differences between the two groups at the following time points: 0:00, 01:00, 02:00, 03:00 and 04:00 (all *P* < 0.05).

**Figure 3:**
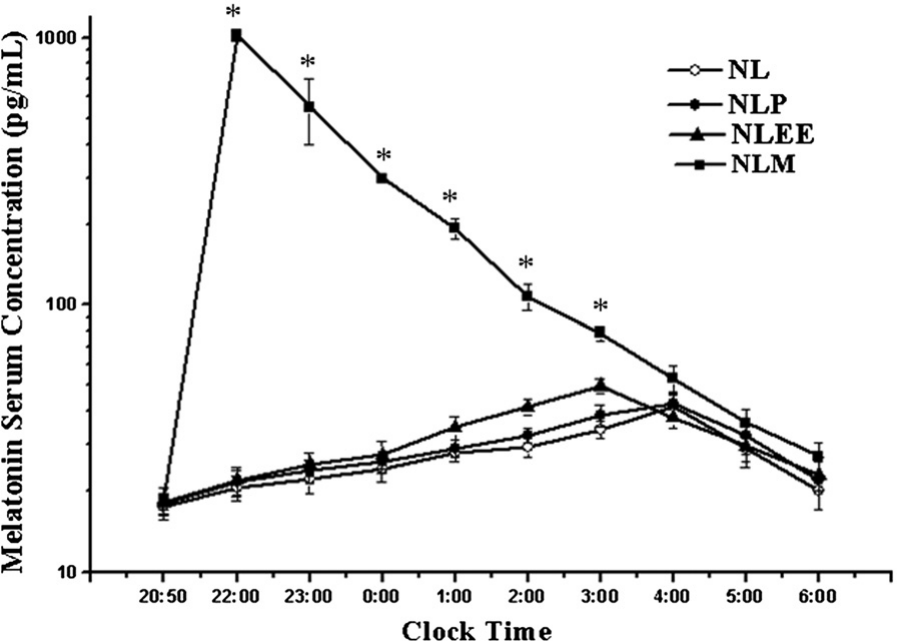
**Serum melatonin concentration time profiles for different study conditions on night 8.** Serum melatonin levels were measured at the end of the study period for 9 h from 20:50 to 06:00. The graph depicts the nocturnal serum melatonin concentration. Points represent mean ± SD. Open circles, healthy subjects on simulated ICU noise and light (NL); solid circles, healthy subjects on NL plus placebo (NLP); solid triangles, healthy subjects on NL plus use of earplugs and eye masks (NLEE); solid squares, healthy subjects on NL plus melatonin (NLM): *P <0.05 at 22:00, 23:00, 0:00, 01:00, 02:00 and 03:00, respectively, for comparison between the NLM and NL nights.

Nocturnal serum melatonin levels during the NL, NLP, NLM and NLEE nights are shown in Figure 3. NL, NLP and NLEE had the similar circadian pattern with the rise in melatonin levels at around 20:50 (before bedtime), reaching peak concentration at 04:00, 04:00 and 03:00, respectively. However, in the NLM group, melatonin was rapidly absorbed following oral ingestion in all the subjects in the NLM group. Maximal melatonin concentration was reached at about 1 h after melatonin treatment, and then the melatonin concentration decreased with time. The average maximal serum concentration (Cmax) in the NLM group was 1021.04 ± 50.58 pg/mL, which was significantly greater than in the other groups (*P* <0.001).

### Subjective sleep quality and anxiety levels

Table 4 shows that compared to the baseline night, simulated ICU noise and light led to worse subjective sleep quality and higher anxiety levels (both *P* = 0.000). The results were significant (all *P* = 0.000) for repeated measures ANOVA for subjective sleep quality and anxiety levels on the simulated ICU noise and light night for different study conditions (Table 5). Paired comparison showed oral administration of melatonin and use of earplugs and eye masks improved perceived sleep quality notably (all *P* = 0.000), and NLM was better than NLEE (*P* = 0.010). No difference was found in anxiety levels between the NLEE and NLM nights (*P* = 0.118) by paired comparison, although SAI scores showed interesting trends towards higher scores for the NLEE night.

**Table 4:** **Subjective sleep quality and state anxiety between baseline and simulated ICU noise and light night**

	**Baseline**	**NL**	***P***
**State anxiety**	29.7 ± 4.3	46.0 ± 8.8	0.000
**Sleep quality**	2.2 ± 1.0	5.7 ± 1.2	0.000

Baseline: quiet and dark environment; NL: simulated ICU noise and light.

**Table 5:** **Subjective sleep quality and anxiety in simulated ICU noise and light night for different conditions**

	**NL**	**NLP**	**NLEE**	**NLM**	**ANOVA**	**Contrast 1**	**Contrast 2**	**Contrast 3**
					***P***	***P***	***P***	***P***
**State anxiety**	46.0 ± 8.8	45.2 ± 7.7	33.5 ± 5.0	28.8 ± 3.3	0.000	0.000	0.000	0.118
**Sleep quality**	6.1 ± 1.0	4.3 ± 1.3	3.4 ± 0.8	2.2 ± 0.8	0.000	0.000	0.000	0.010

NL: simulated ICU noise and light; NLP: NL plus placebo; NLM: NL plus melatonin; NLEE: NL plus use of earplugs and eye masks; Contrast 1: NL vs. NLEE; Contrast 2: NLP vs. NLM; Contrast 3: NLEE vs. NLM.

### Subjects’ evaluations of earplugs and eye masks

Subjects’ evaluations of earplugs and eye masks are listed in Table 6. Overall, most of them rated the earplugs helpful to reduce noise, but uncomfortable and not easy to use. Meanwhile, eye masks were considered to be comfortable, helpful and easy to use.

**Table 6:** **Evaluation of earplugs and eye masks (n = 10)**

		**One**	**Two**	**Three**	**Four**	**Five**
**Earplugs**	**Comfort**	3	4	2	1	0
	**Easy to apply**	1	4	3	1	1
	**Effectiveness**	0	1	3	4	2
**Eye masks**	**Comfort**	0	1	2	5	2
	**Easy to apply**	0	0	1	8	1
	**Effectiveness**	0	1	6	2	1

One: very uncomfortable, very unhelpful, very awkward; Two: uncomfortable, unhelpful, awkward; Three: satisfactory; Four: comfortable, helpful, easy to use; Five: very comfortable, very helpful, very easy to use.

### Safety and tolerance of melatonin

No adverse effects related to the drug were observed in any subject during the study period.

## Discussion

Consistent with previous studies in other samples [6,10,31], we found that nocturnal sleep and body production of melatonin are both disturbed in healthy subjects exposed to simulated ICU noise and light, suggesting our protocol was suitable to test our hypotheses. More importantly, we noted that use of both oral melatonin, and earplugs and eye masks improve sleep quality at different levels, especially melatonin.

In the first part of the study, our results confirmed that in simulated ICU noise and light, healthy subjects not only had greater anxiety and poorer subjective sleep quality, but also suffered from the disturbance of sleep structure, measured as shorter total sleep time, longer sleep-onset latency, longer REM latency, more light sleep, less REM sleep and more arousals and awakenings. Although previous studies have suggested that ICU noise disturbs the sleep quality of healthy subjects to varying degrees [10,31], only the research reported by Hu *et al.* was similar to ours, which combined levels of noise and light in a simulated ICU environment in a sleep laboratory [6].

Although the ICU environment might not be responsible for the majority of ICU sleep disturbance, excessive noise and long-lasting light have proved to be the important and inevitable factors and have negative physiological and psychological effects on patients [9,34]. In some ICUs, a single-bedded room has been chosen for noise reduction, however, most noise leaks from the main ICU [9]. Although mean sound and mean maximum sound intensities are significantly reduced in a single-bedded room, the frequency of sound spikes remained elevated so there is still a lack of improvement in sleep continuity. Recent emphasis has been on controlling noise level and encouraging the dimming of lights overnight in ICU settings, but control of noise is not always possible, and lights are always present in critical care for patient observations and patient care activities [12]. Therefore, some domestic and international experts recommend that the ICU incorporates earplugs/eye mask into routine nursing care [13]. In the second part of our study, consistent with previous studies, use of earplugs and eye masks significantly improved the sleep quality, as seen in shorter sleep-onset latency, reduced number of arousals and awakenings, better perceived sleep quality and less anxiety. But the tolerability of this intervention is critical. Previous studies showed that some ICU patents were unwilling to use the earplugs and/or eye masks because they found the intervention uncomfortable [28]. In our study, most subjects who wore earplugs and eye masks considered this strategy was effective, however, the discomfort of earplugs is a big problem. Some patients commented that there a feeling of tightness, sore ears, claustrophobia and still being able to hear when using earplugs. So it is necessary to explore other methods that are better tolerated and even more effective.

An ideal sleep-improvement strategy for avoiding disruption of the ICU environment should be economical, feasible, rapid in onset and offset, and without local or systemic adverse effects. Recently, melatonin has raised concerns among ICU experts. Melatonin is a key circadian regulatory neurohormone mainly secreted by the pineal gland. Light signals play the most important role in the synthesis and secretion of melatonin in the organism via the retina and retina-hypothalamic pathways, acting directly on the suprachiasmatic nuclei (SCN). So melatonin secretion normally increases at night and decreases in the early morning hours. The melatonin rhythm functions to synchronize circadian rhythms, whereas the melatonin rhythm along with all other circadian rhythms are synchronized by the central pacemaker. Therefore, melatonin is a good sleep aid. The interest in melatonin as a potential therapeutic or prophylactic agent in management of sleep disturbance in the ICU derives from demonstrated low plasma concentrations and altered secretion patterns of melatonin in the critically ill patients [23,24,27]. However, the physiological regulation of melatonin secretion by darkness and light is abolished in severely ill patients in ICU [14], so use of eye masks or dimming the lights only might be not enough to return to normal levels. Thus, supplementation of exogenous melatonin, to remodel the melatonin level in the human body that approaches the physiological state, might be a potential strategy for improving sleep among ICU patients.

The second part of our study investigated and compared the effect of oral melatonin and use of earplugs and eye masks on sleep quality and serum melatonin level in a simulated ICU noise and light environment. Both earplugs and eye masks, and oral melatonin, significantly improved sleep quality, in addition to shorter sleep-onset latency and reduced number of arousals and awakenings; melatonin also increased the duration of REM sleep stage and total sleep time. Furthermore, when oral melatonin was compared to wearing earplugs and eye masks, there was a significant decrease in awakenings and arousals during simulated ICU noise and light. The encouraging results were established both by PSG and by participants’ self-reports and showed that sleep in the simulated ICU noise and light environment while wearing earplugs and eye masks, or after taking melatonin, was more restorative and less fragmented. Although there is no consensus on the relative functions of the various sleep stages, REM sleep generally is considered to be important for cognitive restoration [35]. On the other hand, both awakenings and arousals, and acoustic events, can accelerate the heart rate, which is linked to adverse cardiovascular events and may impair the patient’s recuperation [36-38]. Compared to earplugs and eye masks, melatonin administration might benefit sleep better by three mechanisms: (1) although earplugs can lower noise at a certain degree, the frequency of sound spikes remained elevated, which is an important contributor to noise-induced sleep disruption. However, melatonin has some degree of sedative effect [39] and might buffer the stress from noise. That is probably why in our study melatonin administration without any intervention for attenuating ambient noise improved sleep under the noisy overnight sleep conditions; (2) some patients considered the discomfort of earplugs and eye masks to affect sleep quality; (3) wearing earplugs and eye masks could not make the declined melatonin level return to the normal level, but melatonin supplements could bring a them to a higher level.

Although the cause of the low melatonin level in ICU patients is still unclear, ICU noise and illumination should be significant factors. In the study of Hu *et al*. simulated nocturnal ICU noise and light caused low urinary secretion levels of 6-sulphatoxymelatonin (6-SMT) and high urinary cortisol during the night, and earplugs and eye masks elevated the nocturnal levels of 6-SMT, which suggests that raising the melatonin level might be one of the mechanisms for improving sleep quality [6]. However, metabolites of melatonin cannot entirely represent the true level in the body [40].

There has been no study on the impact of ICU noise and light on nocturnal serum melatonin levels. In normal physiological conditions, the melatonin peaks between 02:00 and 04:00, and troughs during daytime. The average peak melatonin concentration at night is 60 pg/mL, which gradually declines to trough levels of 10 pg/mL during the daytime [15]. Our results for the baseline night were similar to those values. In our study, ICU noise and light significantly suppressed the melatonin level and delayed the peak of melatonin, which also fits with the retrospective study by Jamie *et al*., who found that even small changes in ordinary light exposure during the evening can significantly affect both plasma melatonin concentrations and the entrained phase of the human circadian pacemaker [41]. We also found that although the subjects were exposed to light overnight, the nocturnal melatonin level still maintained a certain rhythm. Some investigators have speculated that the regulation of melatonin secretion by direct control by the environmental light-dark cycle has conferred on humans an evolutionary advantage [42].

In our study, we found that administration of 1 mg of fast-release melatonin was successful in achieving good absorption. The mean maximal serum melatonin concentration was reached at about 1 h after administration, which was close to 20 times higher than the NL-night levels and 12 times higher than baseline levels, and then the levels fell but still remained at a higher level than on the NL, NLP and NLEE nights. Although oral melatonin might result in 10 to 100 times the normal peak night-concentration after ingestion, it has a wide safety margin [29]. It is theoretically possible that high levels of melatonin were achieved with a high dose at night, so that even during the daytime, melatonin blood levels were sufficiently high to promote diurnal sleep as well [29]. Daytime sleep would have then occurred and made nocturnal sleep more difficult. In addition, controlled release formulations would bring an increased risk of prolonged periods of supraphysiologic melatonin levels [43], therefore, we chose a relatively low dose of fast-released melatonin for our study. However, after melatonin administration the melatonin levels were lower than those in previous studies, perhaps because of overnight illumination exposure. The most suitable dose of melatonin in critically ill patients in ICU should be confirmed further, because Mistraletti *et al*. found that after administration of indole the melatonin peak was reached earlier in ICU patients than in healthy volunteers, and the rate of melatonin disappearance was slightly slower [44]. In our study, using earplugs and eye masks during the simulated ICU noise and light elevated melatonin level to some degree and advanced the timing of the clock. However, the melatonin level was much lower than in the melatonin group and that is one of the reasons why melatonin has greater benefit on sleep in the simulated ICU environment.

Our study design has some limitations, which should be reviewed. First, the study was performed in a sleep laboratory with healthy subjects rather than in an ICU setting of critically ill patients, and therefore could not completely simulate the full auditory and visual experience of the ICU. Second, the study was only performed for a 9-h nocturnal period rather than over 24 h. The ICU patients experience circadian rhythm disturbances with sleep traversing the day and night. Therefore, an ideal study should measure the sleep in healthy volunteers lying recumbent over a 24-h period to completely simulate the ICU scenario. In addition, our sample sizes were small, which limited the power of our statistical analyses. Future studies with larger numbers and greater diversity of participants would likely support these recommendations. Finally, although no adverse effects related to the oral melatonin were observed in our healthy subjects during the study period, the potential safety issues related to melatonin administration in ICU patients need to be considered, and the impact of oral melatonin on critically ill patients with ICU sleep deprivation will be our next study focus.

## Conclusions

In summary, our results found that use of melatonin, and earplugs and eye masks in healthy subjects in a simulated ICU environment not only improved subjective sleep quality, but also improved the sleep structure, and elevated nocturnal melatonin levels. Our pilot study provides a reasonable basis for promoting the use of oral melatonin, and earplugs and eye masks for ICU patients. However, compared to earplugs and eye masks, melatonin showed better performance in effectiveness and patient tolerability.

## Key messages

- Nocturnal sleep and body production of melatonin are both disturbed in healthy subjects with exposure to simulated ICU noise and light
- Both oral melatonin and use of earplugs and eye masks improve sleep quality at different levels, especially melatonin
- Both oral melatonin and use of earplugs and eye masks during simulated ICU noise and light elevates melatonin levels to some degree, and advances the timing of the clock. Melatonin is found to be much more effective.
- The discomfort of earplugs and eye masks might affect sleep quality, while no adverse effects of oral melatonin have been observed in any patient during the study period. Therefore, compared to earplugs and eye masks, melatonin shows better performance in effectiveness and patient tolerability.

## Abbreviations

6-SMT: 6-sulphatoxymelatonin
ANOVA: analysis of variance
Baseline_NL_: baseline data in simulated ICU environment
BMI: body mass index
Cmax: maximal serum concentration
CV: coefficients of variation
EEG: electroencephalogram
EMG: electromyogram
NL: simulated ICU noise and light
NLEE: simulated ICU noise and light plus use of earplugs and eye masks
NLM: simulated ICU noise and light plus melatonin
NLP: simulated ICU noise and light plus placebo
NREM: none-rapid eye movement
PSG: polysomnography
PSQI: Pittsburgh sleep quality index
REM: rapid eye movement
RIA: radioimmunoassay
SAI: state anxiety inventory
SCN: suprachiasmatic nuclei
SICU: surgical ICU
SW: slow wave
